# Herbal Formula IM‐B Possesses Antioxidant Activities and Reduces Expression of Proinflammatory Cytokines in Lipopolysaccharide‐Stimulated RAW 264.7 Macrophages

**DOI:** 10.1155/sci5/8315942

**Published:** 2026-06-25

**Authors:** Nuttikarn Nokkaew, Ngamrayu Ngamdokmai, Kittikun Viwatpinyo, Pipob Suwanchaikasem, Woraanong Prugsakij, Parnthep Pourpongpan, Sakan Warinhomhoun

**Affiliations:** ^1^ School of Pharmacy, Walailak University, Thai Buri, Nakhon Si Thammarat, 80160, Thailand, wu.ac.th; ^2^ Herbology Research Center, Walailak University, Thai Buri, Nakhon Si Thammarat, 80160, Thailand, wu.ac.th; ^3^ Faculty of Pharmaceutical Sciences, Burapha University, Chonburi, 20131, Thailand, buu.ac.th; ^4^ Baiya Phytopharm Co., Ltd., Chulalongkorn University, Bangkok, 10330, Thailand, chula.ac.th; ^5^ College of Integrative Medicine, Dhurakij Pundit University, Bangkok, 10210, Thailand, dpu.ac.th; ^6^ College of Oriental Medicine, Rangsit University, Lak Hok, Pathum Thani, 12000, Thailand, rsu.ac.th

**Keywords:** anti-inflammation, antioxidant, herbal formula, IM-B formula, RAW 264.7 macrophage cells

## Abstract

IM‐B is a herbal formula comprising extracts derived from four medicinal herbs previously reported to exhibit antioxidant and anti‐inflammatory properties. In this study, the bioactive compounds of IM‐B extract, as well as its antioxidant and anti‐inflammatory properties, were explored. Chemical compounds in IM‐B extract were identified through phytochemical analysis using LC–ESI–QTOF–MS/MS. Fifteen compounds comprising naringenin 5‐methyl ether, alpinetin, pinocembrin, 2′,4′,6′‐trihydroxydihydrochalcone, demethoxycurcumin, curcumin, alpinetin methyl ether, caffeic acid, myrcene, bisdemethoxycurcumin, geraniol, pinostrobin, pinostrobin chalcone, cannabidiol, and astaxanthin were analyzed. Based on the total phenolic (106.20 ± 3.11 mg GAE/g extract) and flavonoid (94.65 ± 2.65 mg QE/g extract) contents of the IM‐B extract, we consider it a good source of these secondary metabolites. Its antioxidant activities were 4.03 ± 0.63 μg/mL, 9.11 ± 0.57 μg/mL, and 126.84 ± 0.30 mg FeSO_4_/g extract based on 2,2′‐azino‐bis(3‐ethylbenzothiazoline‐6‐sulfonic acid) (ABTS), 2,2‐diphenyl‐1‐picrylhydrazyl (DPPH), and ferric reducing antioxidant power (FRAP) assays, respectively. Analyses of the association of IM‐B extract with nitric oxide (NO), prostaglandin E2, and cytokines (tumor necrosis factor‐*α* [TNF‐*α*] and interleukin‐6 [IL‐6]) revealed that IM‐B extract significantly inhibits the LPS‐stimulated proinflammatory responses in RAW 264.7 macrophage cells. These findings support the potential effectiveness of IM‐B as an herbal supplement in health promotion.

## 1. Introduction

As life expectancy is globally increasing, there is a growing interest in research on diseases and health issues, along with an increasing demand for a better quality of life. Most diseases are linked to imbalanced homeostasis and long‐term inflammation, making oxidative stress and inflammation significant health concerns. It is commonly known that oxidative stress occurs when there is an imbalance between the production of reactive oxygen species (ROS) and the ability of cellular antioxidant defenses to neutralize them [1]. Excessive production of ROS harms cellular macromolecules, and this disruption leads to dysregulated cellular signaling and contributes to the development and progression of inflammatory diseases [2]. While low‐level oxidative stress and inflammation are usual consequences of normal cellular processes in all tissues and organs, dysregulated levels of free radicals and prolonged tissue inflammation can lead to chronic systemic diseases like diabetes, cancer, degenerative diseases, and obesity, as well as speed up the aging process [3]. Therefore, effective control of oxidative stress and inflammation is crucial for preventing the progression of chronic diseases. Among immune cells that are directly involved in the inflammatory process, macrophages are crucial modulators and effector cells of both acute and chronic immune responses, playing vital roles in the initiation, maintenance, and resolution of inflammation [4]. Inflammatory stimuli activate macrophages, which in turn enhance the production of several mediators of acute inflammation, including nitric oxide (NO) and prostaglandin E2 (PGE2), as well as cytokines such as tumor necrosis factor‐alpha (TNF‐α), interleukin‐1 (IL‐1), and interleukin‐6 (IL‐6) [4, 5]. Among diverse types of proinflammatory cytokines, TNF‐α, IL‐6, and PGE2 are considered central mediators in the inflammatory responses and have been associated with the pathogenesis of many diseases [6–8]. Since TNF‐α and IL‐6 stimulate the synthesis of PGE2, which in turn modulates TNF‐α levels these three cytokines create a feedback loop that potentially contributes to mechanisms of chronic diseases, such as arthritis and autoimmune diseases [6, 9, 10]. Therefore, the regulation of TNF‐α, IL‐6, and PGE2 expressions is crucial for the development of novel therapeutic approaches for chronic inflammatory diseases. To investigate these mechanisms, a cellular inflammatory model of the RAW 264.7 macrophage cell line stimulated with lipopolysaccharide (LPS), an endotoxin produced from Gram‐negative bacteria, was established and widely studied [11]. While many immunomodulatory drugs have been developed to regulate the immune responses, their effectiveness is often limited or undesirable, and some have been withdrawn from the market. Thus, there is an ongoing demand to develop new agents that can effectively control or enhance the immune response.

Herbal formulations that are known to possess immunomodulatory effects have been medically used for centuries in the treatment of various human diseases. Increasing evidence suggests that the clinical effects of herbal immunoregulatory formulations are linked to their ability to modulate immune responses, specifically by regulating macrophage cytokine production [12]. Despite extensive investigations about the immunological stimulatory effects of herbal formulations, there is a scarce amount of research relating to the effectiveness of multifunctional phytochemicals in the modulation of the immune system. Several species of medicinal herbs, such as *Haematococcus pluvialis*, *Boesenbergia rotunda*, *Curcuma longa*, and *Cannabis sativa*, are commonly used to treat various inflammatory diseases owing to their antioxidant [13, 14], anticancer [15, 16], and immunomodulatory [17, 18] properties. *B*. *rotunda* exhibits antibacterial [19, 20], anti‐inflammatory [21], antioxidation, and wound recovery properties [22]. The main bioactive compounds in curcuminoids (curcumin, demethoxycurcumin, and bisdemethoxycurcumin) in *C*. *longa* serve as antidiabetic [23], anticancer [24], anti‐inflammatory, antioxidant [25, 26], and immunomodulatory [27] agents. Previous studies have revealed the antioxidative [28], anti‐inflammatory [28–30], and immunomodulatory [31, 32] activities of cannabidiol (CBD), a major compound in *C*. *sativa*.

Hence, in the present study, we prepared an herbal formulation, IM‐B, using *H. pluvialis*, *B. rotunda*, *C. longa*, and *C. sativa* and aimed to evaluate antioxidant activities and the immunoregulatory effects of IM‐B extract in RAW 264.7 murine macrophage cells. The cytotoxicity and immunoregulatory effects of IM‐B extract were determined using cell viability and NO assays. The immunoregulatory activity was explored by assessing the extracellular production of selected cytokines, including TNF‐*α*, IL‐6, and PGE2.

## 2. Materials and Methods

### 2.1. Chemicals and Reagents

IM‐B (a combination of *H. pluvialis* extract, *B. rotunda* extract, *C. longa* extract, and CBD extracted from *C. sativa*) was provided by DR.CBD Co., Ltd. The components of the IM‐B formula are listed in Table 1. Folin–Ciocalteu’s reagent, 2,2′‐azino‐bis(3‐ethylbenzothiazoline‐6‐sulfonic acid) (ABTS), 2,2‐diphenyl‐1‐picrylhydrazyl (DPPH), 3‐(4,5‐dimethylthiazol‐2‐yl)‐2,5‐diphenyl‐tetrazolium bromide (MTT), LPS, gallic acid, quercetin, and other reagents were purchased from Sigma‐Aldrich (St. Louis, MO, USA). Griess reagent was purchased from Promega Co., Ltd. The mouse IL‐6 ELISA (enzyme‐linked immunosorbent assay) kit (Lot CK5E14B) and the mouse TNF‐α ELISA kit (Lot CK5E65C) were provided by MyBioSource Inc. A PGE2 ELISA kit (Lot 09292020D) was purchased from Enzo Life Sciences, Inc. Dulbecco’s modified Eagle’s medium (DMEM) was purchased from the American Type Culture Collection (ATCC, Manassas, VA, USA).

**TABLE 1:** List of ingredients in IM‐B formulation.

Scientific names	Part used	Amount (mg)
*H. pluvialis*	Whole plant	60.00
*B. rotunda* (L.) Mansf.	Rhizome	50.00
*C. longa* L.	Rhizome	25.00
Cannabidiol extracted from *C. sativa*	Flower	0.037

### 2.2. Liquid Chromatography (LC) Analysis of IM‐B Extract

The IM‐B extract was analyzed using LC coupled to an electrospray ionization quadrupole time‐of‐flight mass spectrometer (LC–ESI–QTOF–MS/MS) (Agilent LC‐QTOF 6545XT, Agilent Technologies Inc., Santa Clara, CA, USA) with an ESI source. LC separation was conducted on a Poroshell 120 EC‐C18 (2.1 × 100 mm, 2.7 μm) at 50°C. The IM‐B extract (20.0 mg) was dissolved in methanol containing 25 ng/mL of sulfadimethoxine as an internal standard to a final concentration of 5.0 mg/mL. After that, the samples were filtered through a nylon filter with a pore size of 0.22 μm and centrifuged at 14,000 rpm for 10 min. The gradient mobile phase consisted of a mixture of solvent A (0.1% formic acid in water) and solvent B (0.1% formic acid in acetonitrile) at a flow rate of 0.4 mL/min. The gradient program was initiated with 100% solvent A for 0.5 min, then ramped to 45% solvent A and 55% solvent B for 2 min, then ramped to 25% solvent A and 75% solvent B for 1.5 min, and then ramped to 100% for 1.5 min and automatically returned to the initial mobile phase ratio for 2.5 min. The detection wavelength was set at 280 nm. The injection volume was 10 μL. The total run time was 20 min.

MS analysis was conducted in positive and negative modes with a mass range of 100–1700 m/z. MS parameters were set as follows: gas temperature at 325°C, nebulizer at 45 psi, drying gas at 13 L min^−1^, sheath gas temperature at 275°C, sheath gas flow at 12 L min^−1^, nozzle voltage at 500 V, fragmentor voltage at 175 V, skimmer voltage at 65 V, and capillary voltage at 4000 V and 3000 V for positive and negative modes, respectively. Acquisition time was 3.35 spectra per s. Maximum 10 precursor ions per cycle were selected for MS/MS fragmentation. Collision energy (CE) was 20 and 10 eV for positive and negative modes, respectively. The trifluoroacetic acid (TFA) anion, purine, and HP‐0921 (Agilent Technologies, part no. G1969‐85001) were used as reference compounds. The reference mass was 121.0509 and 922.0098 m/z for positive mode and 112.9596 and 1033.9881 m/z for negative mode. Data were collected in centroid mode. The sample was injected three times.

### 2.3. LC–MS Data Analysis

The raw file was converted to the .abf file format using Reifycs ABF converter with default settings. The converted files were loaded into MS‐DIAL software Version 5.3.24 [33]. Retention time was limited to 0.2–18 min. M + H and M‐H were primarily selected as adduct types for feature detection in positive and negative modes, respectively. Compounds were identified against a combined database available on the MS‐DIAL webpage. The ESI(+)‐MS/MS from authentic standards, Fiehn/Vaniya, and the BMDMS‐NP natural product library were applied [34].

### 2.4. Total Phenolic Contents (TPCs)

The TPC was evaluated using the Folin–Ciocalteu method with some modifications [35]. Extracts (20 μL) were mixed with 100 μL of tenfold‐diluted Folin–Ciocalteu reagent (Sigma‐Aldrich, St. Louis, MO, USA) and 80 μL of sodium bicarbonate (75 g/L) in a 96‐well plate, followed by incubation at room temperature for 1 h. At the endpoint of the reaction, the optical density (OD) of each well was measured at 765 nm with a microplate reader (Thermo Scientific, Göteborg, Sweden). Simultaneously, gallic acid (4–30 μg/mL) was used as a positive control (Sigma‐Aldrich, St. Louis, MO, USA, lot number 099K0128). The results were expressed as milligrams of gallic acid equivalent per gram extract (mg GAE/g extract).

### 2.5. Total Flavonoid Contents (TFCs)

The aluminum chloride colorimetric assay was selected for the assessment of the TFC in the extracts with some modifications [35]. Briefly, 50 μL of extract were mixed with 10 μL of 10% aluminum chloride (Sigma‐Aldrich, St Louis, MO, USA), 10 μL of 1 M sodium acetate (Sigma‐Aldrich, St Louis, MO, USA), and 150 μL of 95% ethanol in a 96‐well plate. The reaction mixtures were subsequently incubated in the dark at room temperature for a further 40 min. The OD of each well was measured at 415 nm using a microplate reader (Multiskan SkyHigh, Thermo Scientific, Göteborg, Sweden) at the endpoint of the reaction. To construct a standard curve for quantification of the TFC, a solution of quercetin (Sigma‐Aldrich, St. Louis, MO, USA, lot number Q4951) in a range of 10–100 μg/mL was made, and the results are expressed as milligrams of quercetin equivalent per gram extract (mgQE/g extract).

### 2.6. DPPH and ABTS Radical Scavenging

The free radical scavenging activity of IM‐B extract was determined using the DPPH assay [36]. Briefly, the sample (1–16 μg/mL) was added to 6.0 × 10^−5^ mol/L DPPH solution in a 96‐well plate. The mixture was then incubated for 30 min at room temperature in the dark. Absorbance was measured at 517 nm using a microplate reader (Multiskan SkyHigh, Thermo Scientific, Göteborg, Sweden). Ascorbic acid concentration at 1.5–24 μg/mL was used as a positive control. The percent scavenging activity (%SA) was calculated using the following equation:

(1) %SA=Ablank−AsampleAblank ×100,

where *A*_sample_ is the absorbance of the DPPH‐treated sample at 517 nm, and *A*_blank_ is the absorbance of DPPH‐treated methanol at 517 nm.

The ABTS assay was conducted according to a previously described method with some modifications [36]. Briefly, 7 mM ABTS (36.0 mg) was dissolved in 10 mL K_2_S_2_O_8_ (2.45 mM), and the solution was incubated at room temperature for 18 h in the dark, resulting in the generation of ABTS^+^ radicals. After 18 h, the ABTS solution was diluted with methanol to ensure a consistent absorbance of 0.71 ± 0.01 at 734 nm. The sample (20 μL) at 2–24 μg/mL was mixed with the solution containing ABTS^+^ radicals (180 μL) in a 96‐well plate. The mixture was then incubated for 30 min at room temperature in the dark. Absorbance was measured at 734 nm using a microplate reader (Multiskan SkyHigh, Thermo Scientific, Göteborg, Sweden). Ascorbic acid at a concentration between 2.5 and 20 μg/mL was used as a positive control. The %SA was calculated using the following equation:

(2) %SA=Ablank−AsampleAblank×100,

where *A*_sample_ is the absorbance of the sample treated with ABTS at 734 nm and *A*_blank_ is the absorbance of ABTS‐treated methanol at 734 nm.

The 50% inhibitory concentration (IC_50_) values of the samples in both DPPH and ABTS assays were determined from a graph plotted against the concentration and percentage of inhibition.

### 2.7. Ferric Reducing Antioxidant Power (FRAP) Assay

The ability of the IM‐B extract to chelate ferrous ions was evaluated using a colorimetric method described in a previous study with some modifications [36]. The FRAP reagent was prepared by mixing 2.5 mL 10 mM TPTZ stock solution, 25 mL acetate buffer (300 mM, pH 3.6), and 2.5 mL 20 mM FeCl_3_ solution (1:10:1). Then, a 50 μL sample (0.5 mg/mL) was mixed with 150 μL FRAP reagent in a 96‐well plate. The mixture was then incubated for 30 min at room temperature in the dark. The absorbance of the intense blue complex formed after the incubation was measured at 594 nm using a microplate reader (Multiskan SkyHigh, Thermo Scientific, Göteborg, Sweden), and the reducing capacity was expressed as mg FeSO_4_/g extract.

### 2.8. Cell Culture

RAW 264.7 macrophage cells obtained from ATCC were cultured in complete media containing DMEM supplemented with 10% FBS, 3.7 g/L sodium bicarbonate, and 1% penicillin–streptomycin in a humidified atmosphere of a 5% CO_2_ incubator at 37°C. After the cells reached 80% confluence, they were removed from the tissue culture flask using trypsin–EDTA.

### 2.9. Viability of RAW 264.7 Macrophage Cells

The effect of IM‐B extract on cell viability of RAW 264.7 macrophage cells was evaluated using the MTT assay. Cells were seeded in 96‐well plates (1 × 10^4^ cells/well) and incubated at 37°C in a humidified atmosphere maintaining 5% CO_2_. After seeding for 24 h, the medium was removed, and the cells were washed using serum‐free media. Subsequently, cells were incubated with IM‐B extract at the concentration range of 25–500 μg/mL in a serum‐free media for 24 h. Next, 100 μL of MTT working solution (1.0 mg/mL in PBS) was added to each well and incubated for 1 h at 37°C and 5% CO_2_. Subsequently, the formed formazan was dissolved in 100 μL of DMSO, followed by measurement of the absorbance at 595 nm using a microplate reader (Multiskan SkyHigh; Thermo Scientific, Göteborg, Sweden). Cell viability was expressed as the percentage of OD of treated cells relative to untreated control cells.

### 2.10. Nitrite Assay

RAW 264.7 macrophages (2 × 10^5^ cells/well) were pretreated with various concentrations (25–200 μg/mL) of IM‐B extract for 1 h and stimulated for 18 h with or without LPS (1.0 μg/mL). Based on the Griess reagent assay, we detected nitrite concentration. An aliquot (50 μL) of the supernatant was incubated with 50 μL of sulfanilamide solution for 10 min. Subsequently, 50 μL of NED solution was added and incubated at room temperature for 10 min protected from light. Nitrite concentration was then assessed by measuring absorbance at 520 nm using a microplate reader (Multiskan SkyHigh, Thermo Scientific, Göteborg, Sweden).

### 2.11. Enzyme‐Linked Immunosorbent Assay (ELISA)

RAW 264.7 macrophages (2 × 10^5^ cells/well) were pretreated with various concentrations (25–200 μg/mL) of IM‐B extract for 1 h and then stimulated for 18 h with or without LPS (1.0 μg/mL). Subsequently, the supernatants were collected, and the amounts of cytokines of interest in the supernatants were quantified using sandwich ELISA assays for TNF‐*α* (MBS825075), IL‐6 (MBS2023471), and PGE2 (ADI‐900‐001), according to the manufacturer’s protocols. Briefly, standards and supernatants were transferred into wells, followed by adding biotinylated antibodies against specific cytokines for the recommended time. After washing away unbound antibodies, horseradish peroxidase (HRP)‐conjugated streptavidin was added to each well, followed by substrate solution and stop solution. The OD of TNF‐*α* and IL‐6 were measured at an absorbance of 450 nm, while PGE2 was measured at 405 nm using a microplate reader (Multiskan SkyHigh, Thermo Scientific, Göteborg, Sweden). Standard curves were created to calculate cytokine concentrations based on the measured OD.

### 2.12. Statistical Analysis

All data derived from three independent experiments were recorded and expressed as mean ± standard deviation (SD). Statistical analyses were performed using GraphPad Prism 9.3.1 (GraphPad Software Inc., San Diego, California, USA) based on one‐way ANOVA and Dunnett’s multiple comparisons as post hoc test. Differences were considered statistically significant at *p* < 0.05.

## 3. Results

### 3.1. Structural Elucidation of the IM‐B Extract

The qualitative analysis of compounds in the IM‐B extract was performed using LC–ESI–QTOF–MS/MS in positive and negative mode. Their structures were inferred from mass spectra and fragmentation patterns. Table 2 lists the negative and positive molecular ions and tentative identification of the 15 detected compounds. The results revealed chemical constituents known to be associated with antioxidant and anti‐inflammatory activities, comprising carotenoids, flavanones, phenolics, curcuminoids, terpenoids, and essential oils.

**TABLE 2:** Compounds identified in the IM‐B extract by LC–ESI–QTOF–MS/MS.

No.	RT (min)	m/z	Error (ppm)	Adduct	MS/MS	Tentative identification (MSI level 2)	Formula	Molecular weight	Group of compounds
1	5.8290	285.0750	6.42	[M − H]^−^	285.0392, 165.0189, 163.0032, 119.0500	Naringenin 5‐methyl ether	C_16_H_14_O_5_	286.28	Flavonoid

2	7.7840	271.1049	−30.95	[M + H]^+^	271.0963, 167.0359, 152.0106, 131.0490	Alpinetin	C_16_H_14_O_4_	270.28	Flavonoid

3	9.4110	257.0861	−20.07	[M + H]^+^	257.0806, 153.0181, 131.0490, 103.0543	Pinocembrin	C_15_H_12_O_4_	256.25	Dihydroxyflavone

4	9.4680	257.0824	−1.91	[M − H]^−^	257.0811, 213.0915, 125.0242, 52.2421	2′,4′,6′‐Trihydroxydihydrochalcone	C_15_H_11_O_4_	258.27	Chalcones

5	9.4430	339.1228	0.09	[M + H]^+^	255.1016, 223.0757, 177.0545, 147.0439	Demethoxycurcumin	C_20_H_18_O_5_	338.40	Beta‐diketone
	9.5980	337.1069	3.71	[M − H]^−^	337.1068, 217.0495, 145.0295, 119.0498				

6	9.6820	369.1348	−4.06	[M + H]^+^	285.1123, 257.0857, 177.0546, 145.0283	Curcumin	C_21_H_20_O_6_	368.39	Beta‐diketone
	9.6170	367.1204	−4.66	[M − H]^−^	367.1176, 217.0500, 175.0398, 149.0605				

7	9.7340	285.1124	−3.23	[M + H]^+^	285.1121, 181.0495, 131.0490, 103.0549	Alpinetin methyl ether	C_17_H_16_O_4_	284.31	Flavonoid

8	9.7380	181.0459	2.26	[M + H]^+^	181.0492, 166.0258, 138.0312, 95.0494	Caffeic acid	C_9_H_8_O_4_	180.16	Hydroxycinnamic acid

9	9.7680	137.1324	−17.87	[M + H]^+^	122.0365, 95.0853, 81.0701, 67.0543	Myrcene	C_10_H_16_	136.23	Monoterpene

10	9.7800	309.1126	−8.38	[M + H]^+^	225.0911, 147.0440, 131.0489, 107.0491	Bisdemethoxycurcumin	C_19_H_16_O_4_	308.30	Beta‐diketone
	9.7830	307.0974	−12.90	[M − H]^−^	307.0962, 187.0396, 143.0499, 119.0501				

11	10.3100	177.1266	19.25	[M + Na]^+^	149.0594, 145.0283, 117.0332, 89.0385	Geraniol	C_10_H_18_O	154.25	Monoterpenoid

12	10.7510	271.0964	0.22	[M + H]^+^	167.0338, 152.0107, 131.0493, 103.0539	Pinostrobin	C_16_H_14_O_4_	270.28	Monohydroxy flavone

13	11.6770	271.0966	−2.36	[M + H]^+^	271.0965, 167.0339, 131.0490, 103.0543	Pinostrobin chalcone	C_16_H_14_O_4_	270.28	Chalcones

14	13.4350	315.2396	−24.39	[M + H]^+^	315.2316, 259.1689, 193.1220, 135.1166	Cannabidiol	C_21_H_30_O_2_	314.50	Cannabinoid
	13.4110	313.2155	5.65	[M − H]^−^	313.2154, 245.1532, 179.1075, 49.2569				

15	14.7150	597.3921	2.76	[M + H]^+^	597.3889, 215.1430, 173.1335, 147.1166	Astaxanthin	C_40_H_52_O_4_	596.80	Carotenone

Abbreviation: MSI, Metabolomics Standards Initiative.

### 3.2. TPC and TFC of IM‐B Extract

The TPC and TFC of the IM‐B extract were evaluated using Folin–Ciocalteu reagent and aluminum chloride, and the results are summarized in Table 3. The TPC was calculated using the regression equation of the calibration curve (*Y* = 0.0043*x* + 0.0379; *R* ^2^ = 0.9993) and expressed as mg GAE/g extract. Similarly, TFC was reported as mg QE/g extract (*Y* = 0.0067*x* + 0.052; *R* ^2^ = 0.9995). The results revealed that the TPC and TFC of the IM‐B extract were 106.20 ± 3.11 mg GAE/g extract and 94.65 ± 2.65 mg QE/g extract, respectively.

**TABLE 3:** Evaluation of antioxidant activities (DPPH, ABTS, and FRAP assays), TPC, and TFC of IM‐B extract.

Samples	DPPH (IC_50_ μg/mL)	ABTS (IC_50_ μg/mL)	FRAP (mg FeSO_4_/g extract)	TPC (mg GAE/g extract)	TFC (mg QE/g extract)
IM‐B	4.03 *±* 0.63^∗^	9.11 *±* 0.57^∗^	126.84 *±* 0.30	106.20 ± 3.11	94.65 ± 2.65
Ascorbic acid	6.12 ± 0.08	10.51 ± 0.49	—	—	—

*Note:* Results are expressed as means ± SDs, *n* = 3.

^∗^ *p* < 0.05, a significant difference compared to ascorbic acid.

### 3.3. Effect of IM‐B Extract on Free Radical Scavenging and Ferrous Ion Chelating Activities

Since antioxidant activity is one of the major characteristics of phenolic and flavonoid compounds, the capability of the IM‐B extract to reduce free radicals was measured by the DPPH and ABTS assays, and the results were shown in Table 3. The DPPH radical scavenging activity and ABTS radical cation decolorization activity are expressed as IC_50_, presenting the concentration of the extract that inhibits the initial free radical by 50%. A lower IC_50_ value indicates a greater potential of the substance for scavenging DPPH and ABTS. The IC_50_ values of both DPPH (4.03 ± 0.63 μg/mL) and ABTS (9.11 ± 0.57 μg/mL) activities of the IM‐B extract were comparable to those of ascorbic acid (IC_50_ value of DPPH 6.12 ± 0.08 μg/mL and IC_50_ value of ABTS 10.51 ± 0.49 μg/mL). These results suggest that IM‐B extract can serve as a strong radical scavenger. The FRAP assay revealed that IM‐B extract exhibited strong ferrous ion chelating ability (126.84 ± 0.30 mg FeSO_4_/g extract).

### 3.4. Effect of IM‐B Extract on RAW 264.7 Macrophage Cells’ Viability

**FIGURE 1:**
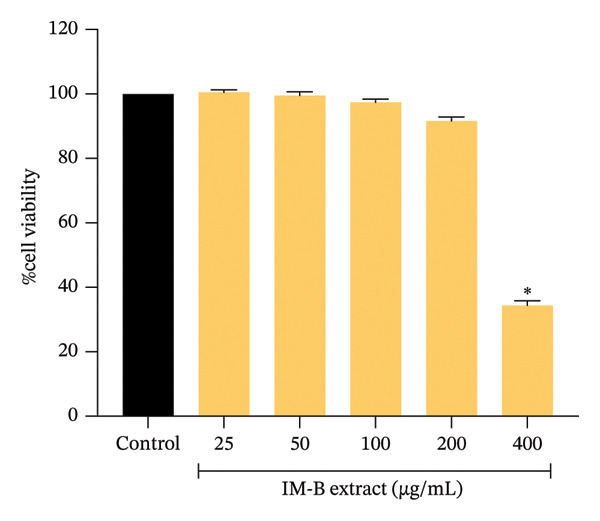
Effect of IM‐B extract on the viability of 264.7 RAW macrophage cells. Cells were treated with IM‐B extract (25–400 μg/mL) for 24 h, and the percentage of cell viability was measured using 3‐(4,5‐dimethylthiazol‐2‐yl)‐2,5‐diphenyl tetrazolium bromide (MTT) assay. The graph presents mean ± SD values of triplicates (*n* = 3). ^∗^ *p* < 0.05 indicates a significant difference compared to the untreated group.

The MTT assay was used to evaluate the cytotoxicity of IM‐B extract on RAW 264.7 cells. The results are reported as a percentage of cell viability, with 100 percent being the untreated control group; IM‐B extract at concentrations of 25, 50, 100, and 200 μg/mL exhibited no cytotoxic effects on cells. However, treatment with 400 μg/mL IM‐B extract significantly (*p* < 0.0001) reduced cell viability compared with the untreated group (Figure 1). Therefore, IM‐B extract concentrations between 25 and 200 μg/mL were used for further study.

### 3.5. Effect of IM‐B Extract on LPS‐Stimulated NO Production and Expression of PGE2 in LPS‐Stimulated RAW 264.7 Cells

**FIGURE 2:**
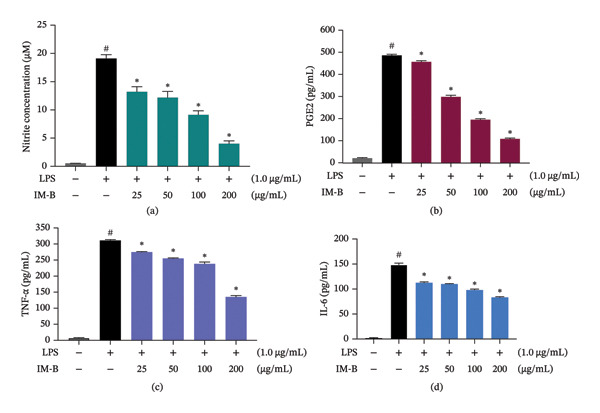
Effects of IM‐B extract on the production of inflammatory mediators and cytokines in LPS‐stimulated RAW 264.7 cells. Cells were pretreated with IM‐B extract (25, 50, 100, and 200 μg/mL) for 1 h followed by an LPS treatment (1.0 μg/mL) for 18 h. (a) Effect of IM‐B on LPS‐induced NO production. The amount of NO produced was measured using the Griess assay. The effect of IM‐B extract on proinflammation (b) PGE2 and cytokines (c) TNF‐*α* and (d) IL‐6 levels was assessed using an ELISA kit. Graphs show the mean ± SD values of data recorded in triplicates (*n* = 3). ^∗^ *p* < 0.05 indicates significant differences from the LPS group. ^#^ *p* < 0.05 indicates significant differences from the untreated group.

**FIGURE 3:**
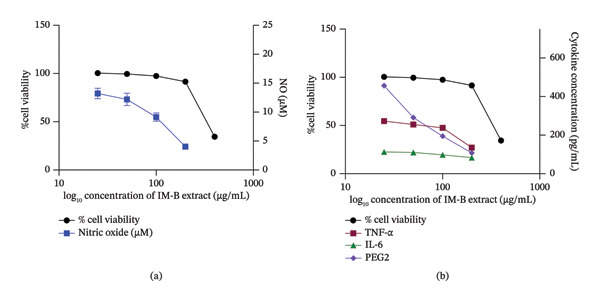
Dose–response cytokine reduction curve of IM‐B extract on RAW 264.7 cells. (a) Dose‐response curve of NO production. (b) Dose–response curve of TNF‐*α*, IL‐6, and PGE2.

The anti‐inflammatory effect of IM‐B extract was evaluated based on NO production in LPS‐stimulated RAW 264.7 cells. The amount of nitrite accumulated in the culture medium was detected using the Griess reagent as an indicator of NO production. The concentration of nitrite in the culture medium increased about 32‐fold (0.54 *±* 0.01 μM vs. 19.09 *±* 0.69 μM) after LPS treatment of cells for 24 h compared to that in the untreated group (Figure 2a). IM‐B extract (25, 50, 100, and 200 μg/mL) significantly (*p* < 0.0001) reduced NO production in a dose‐dependent manner. Next, the anti‐inflammatory effect of IM‐B extract on PGE2 production in LPS‐stimulated RAW cells was evaluated using ELISA (Figure 2b). The results revealed that IM‐B extract significantly (*p* < 0.0001) reduced the expression level of PGE2 at the concentrations of 25 μg/mL (456.83 ± 5.00 pg/mL), 50 μg/mL (298.33 ± 7.15 pg/mL), 100 μg/mL (194.92 ± 4.42 pg/mL), and 200 μg/mL (108.08 ± 3.64 pg/mL) compared with the untreated control counterpart (485.89 ± 5.38 pg/mL). Figure 3a, b illustrates the dose–response relationship between IM‐B extract and the attenuation of NO synthesis, as well as the expression levels of PGE2. The findings suggest that IM‐B effectively diminishes both NO production and PGE2 expression relative to the observed percentages of cell viability.

### 3.6. Effect of IM‐B Extract on the Expression of TNF‐*α* and IL‐6 in LPS‐Stimulated RAW 264.7 Cells

The inflammatory response of macrophage cells to IM‐B extract was investigated by measuring the procytokine release of TNF‐*α* and IL‐6 using ELISA. As shown in Figure 2c, d, IM‐B extract significantly (*p* < 0.0001) downregulated the expression of TNF‐*α* at the concentrations of 25 μg/mL (275.03 ± 1.39 pg/mL), 50 μg/mL (255.04 ± 1.67 pg/mL), 100 μg/mL (238.15 ± 5.60 pg/mL), and 200 μg/mL (135.18 ± 4.35 pg/mL) compared with that observed in the untreated group (310.88 ± 2.87 pg/mL). Additionally, the IM‐B extract significantly (*p* < 0.0001) downregulated the expression of IL‐6 at 25 μg/mL (112.64 ± 1.67 pg/mL), 50 μg/mL (110.06 ± 0.84 pg/mL), 100 μg/mL (98.07 ± 1.82 pg/mL), and 200 μg/mL (83.40 ± 1.18 pg/mL) compared with the untreated counterpart (148.65 ± 3.15 pg/mL). To confirm that the reduced expression levels of the cytokines TNF‐*α* and IL‐6 were not attributable to decreased cell viability, a dose–response curve was evaluated. The findings indicated that IM‐B reduces NO production as well as PGE2 expression (Figure 3b).

## 4. Discussion

This study has demonstrated the effect of the herbal formula IM‐B extract on the antioxidant activities and the production of inflammatory mediators in RAW 264.7 macrophage cells. The results indicate that IM‐B extract effectively inhibits LPS‐stimulated NO, PGE2, TNF‐α, and IL‐6 productions in these cells. Moreover, IM‐B extract was found to be a good source of antioxidants. LC–ESI–QTOF–MS/MS revealed the main bioactive compounds of the IM‐B extract to be phenolics and flavonoids (Table 2). Pinostrobin, pinocembrin, pinostrobin chalcone, alpinetin, alpinetin 5‐methyl ether, naringenin, 2′,4′,6′‐trihydroxydihydrochalcone, geraniol, and myrcene have been reported from rhizomes of *B. rotunda* [37]. Curcumin, bismethoxycurcumin, and demethoxycurcumin were major compounds found in *C. longa* [38]. Previous studies on the HPLC analysis of *C. longa* extract also found caffeic acid [39]. CBD was a chemical constituent in *C. sativa* extract [40]. Astaxanthin was a chemical constituent from *H. pluvialis* [41]. Within the qualitative analysis results, naringenin 5‐methyl ether (RT = 5.8290), alpinetin (RT = 7.7840), pinocembrin (RT = 9.4110), 2′,4′,6′‐trihydroxydihydrochalcone (RT = 9.4680), demethoxycurcumin (RT = 9.4430, 9.5980), curcumin (RT = 9.6820, 9.6170), alpinetin methyl ether (RT = 9.7340), caffeic acid (RT = 9.7380), myrcene (RT = 9.7680), bisdemethoxycurcumin (RT = 9.7800, 9.7830), geraniol (RT = 10.3100), pinostrobin (RT = 10.7510), pinostrobin chalcone (RT = 11.6770), CBD (RT = 13.4350, 13.4110), and astaxanthin (RT = 14.7150) were the main compounds characterized in IM‐B extract. The LC–ESI–QTOF–MS/MS analysis revealed a high content of phenolics and flavonoids, which have strong anti‐inflammation and antioxidant activities.

Extensive research supports the observation that medicinal plants contain significant levels of phenolic and flavonoid compounds, which are recognized for their antioxidant and anti‐inflammatory bioactivities [42, 43]. Our results reflected the antioxidation property of the IM‐B formula. Astaxanthin exhibits strong antioxidant activity [44, 45]. The potential of its antioxidant activity is associated with the β‐ionone rings, keto groups in the positions 4 and 4′ and 3 and 3′, hydroxyl groups in chiral atoms [46], electron transfer radical adduct formation [45], hydrogen atom transfer [45], and redox reactions [47]. Curcuminoids (curcumin, bismethoxycurcumin, and demethoxycurcumin) in *C. longa* extracts are effective antioxidants with evident medicinal effects [48–50]. The number and position of hydroxyl and methoxyl groups and the β‐diketone moiety are mainly associated with the antioxidant action of curcumin; moreover, its derivatives in curcuminoids contribute to the antioxidant properties [51, 52]. The combination of astaxanthin and curcumin decreased the level of ROS, and malondialdehyde (MDA) and increased the antioxidant enzyme catalase (CAT) compared to a single pure compound [53]. Pinostrobin, a bioflavonoid found in various medicinal herbs, exhibits significant antioxidant properties, which contribute to its protective effects in various biological contexts. Its antioxidant activity is primarily demonstrated through the reduction of oxidative stress markers and the enhancement of antioxidant enzyme levels [22, 54, 55]. Moreover, pinocembrin has been reported to reduce oxidative stress linked to its interaction with cellular signaling pathways, particularly the Nrf2/HO‐1 [56–58]. Additionally, CBD was reported to exhibit antioxidant activity [59]. Erukainure et al. [60] reported the antioxidant activity of CBD involves the abstraction of H1 by the radical and delocalization of the radical’s unpaired electron [60]. Therefore, our data imply that the strong inhibition of antioxidant activity by IM‐B extract is potentially attributed to the structure and synergistic effect of its components. It should be noted that we utilized three different antioxidant assays to evaluate the IM‐B extract to explore its antioxidative capabilities in vitro: the DPPH assay for assessing the ability of the extract to donate hydrogen atoms to free radicals in the hydrophobic systems, the ABTS assay for measuring the ability to donate electrons to ABTS+ radical cations in both lipophilic and hydrophilic systems, and the FRAP assay to determine Fe^2+^‐chelating ability and thus prevent free radicals formation via the Fenton reaction [61]. There was also a recent study that utilized these three antioxidant assays together with measurement of NO level [62–66], emphasizing that DPPH, ABTS, and FRAP assays are common methods in the preliminary assessment of antioxidant compounds. However, these antioxidative abilities of IM‐B extract might be altered in the presence of biological factors, such as expression of pro‐oxidative and antioxidative enzymes in inflammatory conditions, and further investigation should include assessment of intracellular antioxidative enzyme activities in the presence of IM‐B extract.

Chronic inflammation is recognized as a substantial concern to human health worldwide, serving as an influential factor in a range of chronic diseases, such as stroke, heart disease, obesity, diabetes, chronic respiratory diseases, and cancer [67]. The increasing demand for phytochemical compounds in inflammatory treatments is associated with the broad spectrum of their biological activities, safety, and reduced risk of serious complications during long‐term usage [68]. This study revealed the anti‐inflammatory activity of IM‐B extract and its efficacy as an alternative treatment to reduce inflammation. LPS, a pivotal component of the outer membrane of Gram‐negative bacteria, is instrumental in provoking an inflammatory response and is responsible for the onset of septic shock in the context of Gram‐negative bacterial infections. [69]. Hence, LPS‐stimulated macrophages are generally employed to assess the anti‐inflammatory properties of different substances [68, 70]. As a key inflammatory mediator, NO contributes significantly to inflammatory responses. Excessive NO production serves as a marker for both acute and chronic inflammatory conditions. The synthesis of NO from L‐arginine is mediated by nitric oxide synthase (NOS) isoenzymes, with inducible NOS (iNOS) being the predominant isoform expressed in activated macrophages. Furthermore, PGE2 is a well‐known mediator of tissue inflammation synthesized from arachidonic acid through the catalytic actions of COX‐1 and COX‐2. During inflammation, cytokines, LPS, and other activators considerably enhance COX‐2 expression and the release of PGE2 at inflammation sites [71]. TNF‐α, a key component of the inflammatory cytokine activation system, possesses the capacity to induce the release of IL‐1β and IL‐6, consequently augmenting the responsiveness of tissue macrophages to TNF‐α stimulation. TNF‐α exhibits fever‐inducing activity and also stimulates endothelial cells and white blood cells to release a cascade of inflammatory mediators, including NO and ROS. This release of mediators can subsequently contribute to enhanced TNF‐α production, creating a potential positive feedback mechanism [71]. A previous report demonstrated an inhibitory effect of PGE2 on TNF synthesis via elevated cAMP levels [72]. Our results revealed that IM‐B extract‐mediated dose‐dependent inhibition of NO and PGE2 production and decreased expression of cytokine and TNF‐α and PGE2 levels (Figure 2a, b, c, d). Astaxanthin has been shown to reduce the production of NO and PGE2 and downregulates the expression of TNF‐α [73, 74] and IL‐6 [75]. Curcuminoid treatment of RAW 264.7 cells also reduces NO, PGE2, TNF‐α, and IL‐6 [76–78]. Pinostrobin has been shown to decrease NO production in inflammatory conditions. In a study involving LPS‐stimulated macrophages, pinostrobin reduced NO levels by inhibiting iNOS expression and also reduced PGE2 production [79]. Pinocembrin has been demonstrated to decrease TNF‐α and IL‐6 levels by inhibiting the phosphorylation of key signaling proteins such as IκBα, ERK1/2, JNK, and p38MAPK [80]. In addition, CBD exhibited inhibitory effects on IL‐6, TNF‐α, and PGE2 [81–84]. A previous study reported that curcuminoids derived from *C. longa* and chalcones from *B. rotunda* predominantly suppress the synthesis of proinflammatory mediators through the modulation of nuclear factors [85–88]. Chaiwangrach et al. [89] revealed that the combination of cannabis and turmeric extract strongly suppressed IL‐1β and NO production [89]. Moreover, the combination effect of astaxanthin and curcumin reduced the expression of IL‐6 levels and IFN‐*γ* compared to single compounds in PCOS mice [53]. Considering the abundance of these bioactive constituents in the fractions obtained in this study, we suggest that the potent anti‐inflammatory effect of IM‐B extract is attributable to the synergistic effects of phytoconstituents such as astaxanthin, curcumin, bismethoxycurcumin, demethoxycurcumin, pinostrobin, pinocembrin, and CBD. While this research demonstrated the promising potential of the herbal formula, its effects were evaluated through biomarker analysis known to be expressed in RAW 264.7 cells. Further studies using optimum doses and comparative functional analyses could clarify the potency and mechanism of action of IM‐B extract such as NF‐κB, iNOS, COX‐2, MAPK, or Nrf2, which are associated with the management of inflammatory disorders in RAW 264.7 cells. To establish a strong foundation for potential health promotion, it is imperative to validate these findings through further investigation in diverse cell lines and appropriate animal models.

## 5. Conclusions

This study demonstrated that the herbal formula IM‐B extract effectively exhibits antioxidant and anti‐inflammatory effects on LPS‐induced RAW 264.7 cells. Its 15 bioactive compounds were analyzed: naringenin 5‐methyl ether, alpinetin, pinocembrin, 2′,4′,6′‐trihydroxydihydrochalcone, demethoxycurcumin, curcumin, alpinetin methyl ether, caffeic acid, myrcene, bisdemethoxycurcumin, geraniol, pinostrobin, pinostrobin chalcone, CBD, and astaxanthin. The results indicate that IM‐B extract can modulate macrophage‐mediated inflammation associated with the proinflammatory mediators (NO and PGE2) and cytokines by suppressing the activation of TNF‐α and IL‐6. Further research can validate its effectiveness as a functional herbal supplement with anti‐inflammatory effects.

## Author Contributions

Sakan Warinhomhoun, Nuttikarn Nokkaew, and Kittikun Viwatpinyo conceptualized the study. Methodology, investigation, and software were performed by Sakan Warinhomhoun, Nuttikarn Nokkaew, Kittikun Viwatpinyo, Ngamrayu Ngamdokmai, Woraanong Prugsakij, and Pipob Suwanchaikasem. Validation, formal analysis, and resources were performed by Sakan Warinhomhoun, Nuttikarn Nokkaew, Ngamrayu Ngamdokmai, and Kittikun Viwatpinyo. The manuscript was written, reviewed, and edited by Sakan Warinhomhoun, Nuttikarn Nokkaew, Kittikun Viwatpinyo, Ngamrayu Ngamdokmai, and Parnthep Pourpongpan. Supervision was performed by Sakan Warinhomhoun and Nuttikarn Nokkaew. Visualization, project administration, and funding acquisition were performed by Sakan Warinhomhoun.

## Funding

We are thankful to Walailak University and DR. CBD Co., Ltd. for supporting this research under a joint project (grant number WUSTP‐13/2565).

## Disclosure

All authors have read and agreed to the published version of the manuscript.

## Conflicts of Interest

The authors declare no conflicts of interest.

## Data Availability

The experimental data used to support the findings of this study are available from the corresponding author upon request.
